# Utilizing Predictive Factors as a Screening Tool for Early-Onset Sepsis in Neonates

**DOI:** 10.7759/cureus.66312

**Published:** 2024-08-06

**Authors:** Nhu Thi Huynh Tran, Ly Cong Tran, Duc Long Tran, Vinh The Nguyen, My Hoang Le, Nhi Thi Kieu Nguyen

**Affiliations:** 1 Department of Pediatrics, Can Tho University of Medicine and Pharmacy, Can Tho, VNM

**Keywords:** vietnam, neonatology, pediatrics, developing nations, prediction tools, resource limited setting, neonatal early-onset sepsis

## Abstract

Introduction: Neonatal early-onset sepsis (EOS) is a severe condition that affects newborns within the first three days of life, with high mortality rates, particularly in low- and middle-income countries (LMICs). In Vietnam, the diagnosis and management of EOS are challenged by ambiguous clinical signs and limited access to blood culture testing facilities. Early identification of at-risk neonates using a predictive risk factor model is crucial for improving neonatal care and reducing mortality.

Objectives: This study aims to identify maternal and neonatal risk factors associated with EOS and develop a predictive screening tool to facilitate the early detection of at-risk neonates in Vietnam.

Materials and methods: A nested case-control study was conducted on 225 neonates at the central neonatal unit in a principal tertiary hospital in southwestern Vietnam over a two-year period. Risk factors were identified using univariable and multivariable logistic regression analyses. A predictive nomogram was developed and evaluated for discrimination, calibration, and decision curve analysis (DCA).

Results: The study identified eight significant risk factors for EOS, including maternal genital infections during the third trimester, urinary tract infections (UTIs) during pregnancy, hypertension during pregnancy, insufficient maternal weight gain, rupture of membranes (ROM) ≥18 hours, meconium-stained amniotic fluid, first-minute APGAR score <7, and preterm birth <34 weeks. The predictive model demonstrated excellent discrimination with an area under the curve (AUC) of 0.913 (95% CI: 0.876-0.95, p<0.001) and good calibration (Hosmer-Lemeshow test with χ²(df)=5.496 (5), p=0.358). The model-based nomogram showed high sensitivity (82.7%) and specificity (83.3%) at an optimal cutoff of 0.25. The DCA illustrates the model's good clinical utility, providing a higher net benefit across most threshold probability ranges (0.0-0.96).

Conclusions: This study presents a robust predictive model for the early identification of neonates at risk of EOS in Vietnam, based on key maternal and neonatal risk factors. The model, with demonstrated accuracy and reliability, holds significant potential for improving neonatal outcomes through timely interventions. Future research should aim at external validation and inclusion of broader clinical data to enhance the model's applicability and generalizability.

## Introduction

Neonatal early-onset sepsis (EOS) is a serious, life-threatening condition that affects newborns within the first three days of life [1]. Mortality rates for EOS can be alarmingly high, reaching up to 30% in high-income countries and 60% in low-income countries [2]. Neonatal sepsis is a major cause of hospitalization and death in low- and middle-income countries (LMICs). In Vietnam, neonatal sepsis is prevalent, with mortality rates ranging from 13.2% to 46% among affected newborns [3].

However, the signs of EOS are often varied, ambiguous, and complex, particularly in preterm neonates [4]. These varied presentations can make early detection of EOS challenging for physicians. Unlike older children, where sepsis signs are more apparent and straightforward to diagnose, neonates exhibit symptoms that overlap with normal physiological adjustments post-delivery, complicating diagnosis. Additionally, neonates can experience bacteremia without exhibiting any clinical signs or symptoms [5,6]. While microbial cultures are essential for pathogen identification and provide information for targeted antimicrobial therapy and duration, their positivity rate is low and is influenced by factors such as the volume of blood inoculated, prenatal antibiotic use, the level of bacteremia, and laboratory capabilities. In developing countries, culture-negative sepsis accounts for the majority of sepsis episodes [7,8]. In Vietnam, this challenge is compounded by the lack of adequate blood culture testing facilities in many hospitals, further hindering effective diagnosis [3].

Understanding the risk factors associated with increased susceptibility to sepsis in neonates is crucial for early diagnosis in this context [9]. In resource-limited settings, where blood culture facilities are often unavailable, a predictive risk factor model can facilitate early diagnosis and effective management of EOS, decreasing mortality rates. Using a risk factor-based approach to guide treatment decisions has alleviated the burden of neonatal sepsis in LMICs [10,11].

Timely care for neonates at risk of EOS immediately after birth is crucial, ensuring these vulnerable newborns receive comprehensive medical attention promptly. However, identifying neonates suspected of sepsis requires experienced pediatricians, a challenge exacerbated by the shortage of physicians in LMICs. Inadequate coordination between obstetric and pediatric care can negatively impact newborn outcomes, particularly during the transitional period [12]. Enhancing this coordination is essential in resource-limited settings where professional physician availability is limited. By employing predictive tools accessible and widely used by obstetricians and midwives, who are present from the moment of birth, at-risk newborns with EOS can be promptly identified. This approach not only conserves human resources but also allows pediatricians to provide targeted, point-of-care treatment for these vulnerable neonates.

Currently, there is a paucity of data on using neonatal sepsis risk factors as a screening tool for predicting EOS in Vietnam. Understanding and utilizing these risk factors is crucial for early identification of the most vulnerable neonates. This early identification is vital to developing effective strategies aimed at improving clinical outcomes. Therefore, this study seeks to identify the risk factors for neonatal EOS and develop a predictive screening tool based on these factors. The goal is to facilitate the early detection of at-risk neonates, thereby enhancing neonatal care and reducing morbidity and mortality rates through timely and targeted interventions.

## Materials and methods

Study design and settings

This nested case-control study was conducted at the Department of Neonatology and Pediatrics at Can Tho Gynecology and Obstetrics Hospital, a leading central obstetric facility in the Mekong Delta, southern Vietnam. The hospital, equipped with extensive neonatal care units, offers tertiary care to a substantial neonatal population. The study spanned a two-year period, from June 2020 to June 2022, and included neonates diagnosed with EOS and those without EOS.

The required sample size was calculated using a two-proportion comparison formula with a two-sided test, a significance level (α) of 5%, and a power of 85%. According to a previous study, the proportion of chorioamnionitis, a risk factor for EOS, was 24.8% in the EOS group and 9.3% in the group without EOS [13]. With a control-to-case ratio of 2:1, the necessary sample size was determined to be 75 in the case group and 150 in the control group, totaling 225 neonates.

Inclusion and exclusion criteria

Neonates aged ≤3 days postnatal were eligible for inclusion in this study. Those diagnosed with EOS by neonatologists were recruited into the case group. In contrast, neonates without signs or a diagnosis of EOS or any infection, born on the same day, and cared for in the same department with similar socioeconomic backgrounds, were randomly selected for the control group. Neonates were excluded if they met any of the following criteria: congenital abnormalities requiring surgery, orphaned or abandoned status, being born at home, or if their mothers were unable to complete the questionnaires for objective reasons.

Operational definition

Cases of neonatal EOS were identified within the first 72 hours of life. Although microbial cultures are essential for pathogen identification and confirmation of sepsis, providing critical information for targeted antimicrobial therapy and duration, definitions in adult and pediatric sepsis have evolved to emphasize pathophysiology without an absolute requirement for positive cultures [7]. At our center, performing microbial cultures is not routine due to resource limitations, a common issue in LMICs. Additionally, the positivity rate of microbial cultures is relatively low, and awaiting results can delay initial treatment in vulnerable neonates. Given these reasons, this study adopted the European Medicines Agency (EMA) 2010 criteria for sepsis diagnosis [14]. According to these criteria, probable sepsis is defined by the presence of ≥2 clinical and ≥2 laboratory criteria.

The clinical manifestations of neonatal sepsis encompass a range of systemic signs. Modified body temperature includes a core temperature >38.5°C or <36°C and/or temperature instability. Cardiovascular instability is characterized by bradycardia (mean heart rate <10th percentile for age without external vagal stimulus, β-blockers, or congenital heart disease, or otherwise unexplained persistent depression over a 0.5-4 hour period), tachycardia (mean heart rate >2 standard deviations above normal for age without external stimulus, chronic unexplained persistent elevation over a 0.5-4 hour period), rhythm instability, reduced urinary output (<1 mL/kg/h), hypotension (mean arterial pressure <5th percentile for age), mottled skin, and impaired peripheral perfusion. Skin and subcutaneous lesions include petechial rash and sclerema. Respiratory instability presents as apnea episodes, tachypnea episodes (mean respiratory rate >2 standard deviations above normal for age), increased oxygen requirements, or the need for ventilatory support. Gastrointestinal symptoms comprise feeding intolerance, poor sucking, and abdominal distension. Non-specific signs include irritability, lethargy, and hypotonia [14].

Laboratory indicators of neonatal sepsis are marked by white blood cell counts <4×10^9^ cells/L or >20×10^9^ cells/L, an immature to total neutrophil ratio (I/T) >0.2, a platelet count <100×10^9^/L, C-reactive protein (CRP) levels >15 mg/L, and procalcitonin levels ≥2 ng/mL. Glucose intolerance is evidenced by hyperglycemia (blood glucose >180 mg/dL or 10 mM) or hypoglycemia (blood glucose <45 mg/dL or 2.5 mM), confirmed at least twice when receiving age-specific normal-range glucose amounts. Metabolic acidosis is indicated by a base excess of less than -10 mEq/L or serum lactate >2 mmol/L [14].

Participant selection and recruitment

In this nested case-control study, both cases and controls were selected from the same department in a central hospital. For each identified case of neonatal sepsis, case-based sampling was used with two controls selected, resulting in a control-to-case ratio of 2:1. To minimize temporal bias, both cases and controls were matched by birthdate. The selection process involved a thorough review of birth records and medical charts to identify all neonates born on the same day as each case. Inclusion criteria required that neonates have no significant differences in socioeconomic status, such as maternal living location (rural or urban), family income, and maternal education levels. From the pool of eligible neonates born on the same day and meeting the socioeconomic criteria, two controls without signs or a diagnosis of sepsis were randomly selected for each case. This random selection was conducted using computer-generated random numbers by an independent third party, ensuring unbiased selection and maintaining the integrity of the study design. All relevant demographic, clinical, and laboratory data were extracted from hospital records for both cases and controls. This approach ensured that study groups were drawn from the same population, thereby enhancing the validity and reliability of the study findings.

Data collection

Data were collected using a questionnaire administered through interviews with mothers and by reviewing the medical records of neonates and their mothers. The outcome variable was neonatal EOS. Independent variables included both maternal and neonatal factors. Maternal characteristics assessed were maternal age, number of pregnancy check-ups, Tdap vaccination during pregnancy, intrapartum fever, multiple gestations, mode of delivery, genital infections in the third trimester, urinary tract infections (UTIs) during pregnancy, chorioamnionitis, hypertension during pregnancy, preeclampsia, gestational diabetes mellitus, pregnancy weight gain, prolonged rupture of membranes (ROM), prolonged labor, and meconium-stained amniotic fluid. Neonatal characteristics included gender, birth weight, gestational age, neonatal classification, intrapartum fetal distress, and APGAR scores at the first and fifth minutes.

Statistical analysis

Quantitative data were expressed as mean (SD) and analyzed using t-tests, while qualitative data were presented as frequency (percentage) and assessed through chi-square or Fisher's exact tests where appropriate. To identify predictors of EOS in neonates, candidate variables were selected through univariable logistic regression, considering those with a p-value <0.05 and presented with odds ratios (OR) and their 95% confidence intervals (CI). These variables were then subjected to multivariable logistic regression using a backward stepwise method based on the Akaike Information Criterion (AIC) to enhance model accuracy and simplicity. A nomogram was developed to visualize the prediction model in a user-friendly manner. The final model was assessed for validation through discrimination, calibration performance, and decision curve analysis (DCA). Discrimination of the prediction model was evaluated using the receiver operating characteristic (ROC) curve and the optimal cutoff determined by the Youden index. Calibration assessment involved evaluating the model’s fit using the Hosmer-Lemeshow test and a calibration curve, with internal validation performed using the bootstrap method with 1000 iterations. The clinical usefulness of the model was evaluated through DCA. All analyses were conducted using R version 4.4.1 (R Foundation for Statistical Computing, Vienna, Austria).

Ethical statement

The ethical and scientific aspects of the research were evaluated and approved by the Ethics Committee in Biomedical Research of Can Tho University of Medicine and Pharmacy on May 28th, 2020 (IRB approval No. 207/PCT-HĐĐĐ). Informed consent was obtained from the parents or legal guardians before conducting the study.

## Results

General characteristics

The study involved 225 newborns, comprising 75 cases and 150 controls, with no missing values for any variables. The comparison of general characteristics between cases and controls is shown in Table 1. There was no significant difference in maternal age between neonates with and without EOS (29.2 years in the case group and 28.8 years in the control group). The rates of low birth weight (LBW) (<2,500g) were 40.0% (30/75) in the case group and 14.7% (22/150) in the control group.

**Table 1:** General Characteristics of the Study Subjects Data are presented as n (%) or mean ± SD, as indicated in each variable row; ^* ^p-value <0.05 was considered significant; ^a ^Chi-square test for independence, with ꭓ^2^-statistic (df) value; ^b ^Fisher’s exact test; AGA: appropriate for gestational age; SGA: small for gestational age; LGA: large for gestational age; SD: standard deviation; APGAR: Appearance, Pulse, Grimace, Activity, and Respiration

Variables	Total (n=225)	Case (n=75)	Control (n=150)	Statistic Value	P-value
Maternal characteristics					
Maternal age (years), mean ± SD	29.1 ± 5.3	29.2 ± 5.2	28.8 ± 5.7	0.57 (136.1)	0.568^a^
Pregnancy check-ups <3 times, n (%)	1 (0.4)	1 (1.3)	0 (0.0)	-	0.333^b^
Pregnancy Tdap vaccination, n (%)	220 (97.8)	71 (94.7)	149 (99.3)	-	0.043^b,*^
Intrapartum fever, n (%)	4 (1.8)	4 (5.3)	0 (0)	-	0.012^b,*^
Multiple gestations, n (%)	11 (4.9)	8 (10.7)	3 (2.0)	-	0.007^b,*^
Mode of delivery, n (%)					
Spontaneous	59 (26.2)	25 (33.3)	34 (22.7)	2.41 (1)	0.12^a^
Cesarean/instrumental	166 (73.8)	50 (66.7)	116 (77.3)		
Neonatal characteristics					
Male gender, n (%)	115 (51.1)	40 (53.3)	75 (50.0)	0.11 (1)	0.741^a^
Birth weights, n (%)					
<1000g	4 (1.8)	4 (5.3)	0 (0.0)	-	<0.001^b,*^
1000-<1500g	5 (2.2)	5 (6.7)	0 (0.0)		
1500-<2500g	43 (19.1)	21 (28.0)	22 (14.7)		
2500-<4000g	169 (75.1)	45 (60.0)	124 (82.7)		
≥4000g	4 (1.8)	0 (0.0)	4 (2.7)		
Gestational age, n (%)					
<34 weeks	29 (12.9)	26 (34.7)	3 (2.0)	-	<0.001^b,*^
34-36 weeks	35 (15.6)	8 (10.7)	27 (18.0)		
37-40 weeks	161 (71.6)	41 (54.7)	120 (80.0)		
≥41 weeks	0 (0.0)	0 (0.0)	0 (0.0)		
Neonatal classification, n (%)					
AGA	203 (90.2)	66 (88.0)	137 (91.3)	-	0.030^b,*^
SGA	8 (3.6)	6 (8.0)	2 (1.3)		
LGA	14 (6.2)	3 (4.0)	11 (7.3)		
Intrapartum fetal distress, n (%)	62 (27.6)	25 (33.3)	37 (24.7)	1.47 (1)	0.225^a^
First-minute APGAR score <7, n (%)	26 (11.6)	23 (30.7)	3 (2.0)	37.45 (1)	<0.001^a,*^
Fifth-minute APGAR score <7, n (%)	1 (0.4)	1 (1.3)	0 (0)	-	0.333^b^

Term neonates accounted for 71.6% (161/225) of the participants, and the majority were classified as appropriate for gestational age (AGA) (90.2%, 203/225). Spontaneous delivery accounted for nearly 26.2% (59/225) of births, while approximately 73.8% (166/225) were delivered via cesarean or instrument-assisted delivery. The APGAR score at the first minute was less than 7 in 30.7% (23/75) of cases and 2.0% (3/150) of controls (p<0.001). There was no significant difference in APGAR scores at the fifth minute after birth. The majority of mothers (99.6%, 224/225) had more than three prenatal examinations. Only a minority of mothers did not receive Tdap vaccination during pregnancy, with four (5.3%, 4/75) in the case group and one (0.7%, 1/750) in the control group. Regarding intrapartum fever, only four (5.3%, 4/75) of case mothers experienced this condition.

Maternal and neonatal predictors of EOS

Table 2 presents the results of univariable logistic regression analyses of risk factors associated with neonatal EOS. Neonates born to mothers with genital infections in the third trimester or UTIs during pregnancy exhibited a 5.81-fold increase in the odds of developing EOS compared to those without these conditions (95% CI, 2.58-13.08; p <0.001 and 95% CI, 3.17-10.66; p<0.001, respectively). Furthermore, neonates born to mothers who gained less than 10 kg during pregnancy had 5.31 times higher odds of developing EOS compared to those who gained more than 10 kg (95% CI, 2.27-12.45; p<0.001). Mothers with hypertension during pregnancy had a 5.61-fold increased risk of EOS (95% CI, 2.31-13.62; p<0.001). Additionally, the odds of EOS were 7.65 times higher among newborns born to mothers with ROM lasting ≥18 hours and 4.46 times higher in those exposed to meconium-stained amniotic fluid (95% CI, 2.4-24.39; p=0.001 and 95% CI, 1.47-13.57; p=0.008, respectively).

**Table 2:** Univariable Logistic Regression Analysis of Risk Factors for Early-Onset Sepsis ^*^p-value <0.05 was considered significant; UTI: urinary tract infections; IDs: instrumental vaginal deliveries; CSs: Caesarean sections; APGAR: Appearance, Pulse, Grimace, Activity, and Respiration; SGA: Small for gestational age; LBW: low birth weight; OR: odds ratio; CI: confidence intervals

Factors	OR	95% CI	Wald Z	P-value
Maternal factors				
Genital infections in the third trimester	5.81	2.58-13.08	4.25	<0.001^*^
UTI in pregnancy	5.81	3.17-10.66	5.69	<0.001^*^
Chorioamnionitis	25.61	3.24-202.54	3.07	0.002^*^
Hypertension during pregnancy	5.61	2.31-13.62	3.81	<0.001^*^
Preeclampsia	11.39	2.43-53.42	3.08	0.002^*^
Gestational diabetes mellitus	2.07	0.58-7.39	1.12	0.262
Pregnancy weight gain <10kg	5.31	2.27-12.45	3.85	<0.001^*^
Multiple gestations	5.85	1.5-22.75	2.55	0.011^*^
Rupture of membranes ≥18h	7.65	2.4-24.39	3.44	0.001^*^
Prolonged labor ≥24h	2.44	1.13-5.26	2.28	0.023^*^
IDs/CSs	1.71	0.92-3.15	-1.71	0.088
Meconium-stained amniotic fluid	4.46	1.47-13.57	2.63	0.008^*^
Neonatal factors				
First minute APGAR score <7	21.67	6.25-75.19	4.85	<0.001^*^
SGA	6.43	1.27-32.69	2.24	0.025^*^
LBW <2500g	4.13	2.19-7.75	4.40	<0.001^*^
Preterm Birth <34w	26.0	7.54-89.67	5.16	<0.001^*^

Infants born before 34 weeks of gestation had 26.0 times higher odds of developing EOS compared to those born after 34 weeks (95% CI, 7.54-89.67; p<0.001). An APGAR score of less than 7 at the first minute after birth was significantly associated with EOS, with an over 21-fold increase in the likelihood of developing EOS (OR=21.67; 95% CI, 6.25-75.19; p<0.001).

Predictive tool and nomogram construction

Using a backward stepwise method in multivariable logistic regression analysis, eight variables were identified as significantly associated with neonatal EOS. The final model, which optimally balances simplicity with enhanced validity and accuracy, is presented in Table 3.

**Table 3:** Multivariable Logistic Regression Analysis of Risk Factors for Early-Onset Sepsis ^*^p-value <0.05 was considered significant; UTI: urinary tract infections; APGAR: Appearance, Pulse, Grimace, Activity, and Respiration; OR: odds ratio; CI: confidence intervals

Factors	Coefficient	OR	95% CI	Wald Z	P-value
Genital infections in the third trimester	1.585	4.88	1.67-14.29	2.89	0.004^*^
UTI in pregnancy	1.901	6.69	2.76-16.24	4.20	<0.001^*^
Hypertension during pregnancy	1.611	5.01	1.30-19.3	2.34	0.019^*^
Pregnancy weight gain <10kg	1.743	5.72	1.62-20.22	2.70	0.007^*^
Rupture of Membranes ≥18h	2.459	11.69	1.97-69.19	2.71	0.007^*^
Meconium-stained amniotic fluid	2.632	13.89	3.42-56.52	3.68	<0.001^*^
First minute APGAR score <7	2.983	19.74	3.56-109.55	3.41	0.001^*^
Preterm birth <34w	3.509	33.43	6.78-164.87	4.31	<0.001^*^
Intercept	-3.340	-	-	-7.55	<0.001^*^

**Figure 1:**
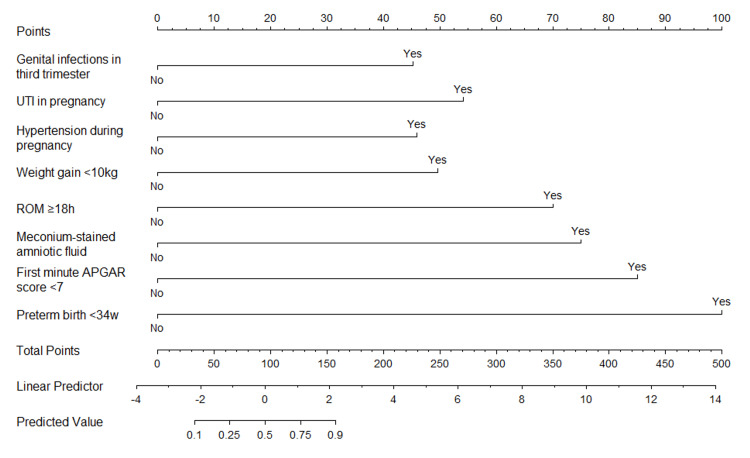
Nomogram for Predicting Early-Onset Sepsis in Neonates UTI: urinary tract infections; ROM: rupture of membranes; APGAR: Appearance, Pulse, Grimace, Activity, and Respiration

A prognostic nomogram was developed to facilitate early identification of neonates at risk of progressing to EOS. This tool was constructed using multivariable logistic regression analysis, which identified eight independent risk factors. These factors were incorporated into an individualized nomogram model for predicting EOS in neonates (Figure 1). Each risk factor was assigned a score ranging from 0 to 100. Specifically, the presence of genital infections in the third trimester was given 45 points, UTIs during pregnancy 54 points, hypertension during pregnancy 46 points, maternal weight gain of less than 10 kg 50 points, ROM lasting 18 hours or more 70 points, meconium-stained amniotic fluid 75 points, an APGAR score below 7 at the first minute 85 points, and preterm birth before 34 weeks 100 points.

By summing these points, a total score is obtained, which can be located on the nomogram scale to determine the risk of EOS at the time of patient presentation. For instance, if a neonate has risk factors such as genital infections in the third trimester and maternal weight gain of less than 10 kg, with no other risk factors, the combined total of 95 points would predict a 50% risk of developing EOS.

This predictive model allows for timely and targeted interventions, potentially improving neonatal outcomes by facilitating early recognition and treatment of those at the highest risk for EOS.

Discrimination and calibration performance of the predictive tool

**Figure 2:**
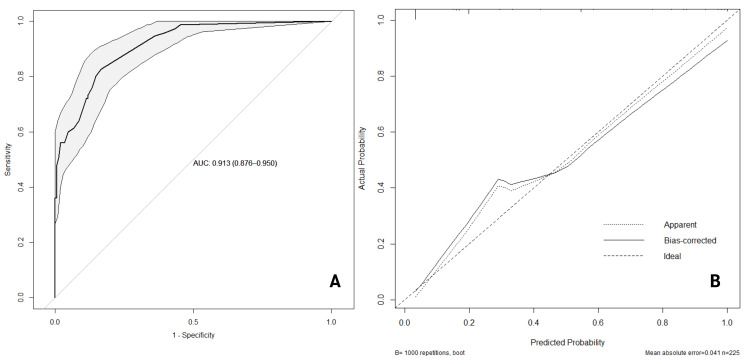
Discrimination and Calibration Performance Through the Receiver Operating Characteristic Curve (A) and Calibration Curve (B) In Figure 2A, the gray area represents the 95% confidence interval range of the receiver operating characteristic (ROC) curve. In Figure 2B, the "Apparent" line shows the in-sample calibration of the model, indicating how well the model's predicted probabilities match the observed probabilities within the sample used to create the model. The "Ideal" line represents perfect prediction, where the predicted probabilities are equal to the observed probabilities. The "Bias-corrected" line is obtained through a resampling procedure, providing an adjusted calibration curve that better represents the model's performance and indicates its generalizability. AUC: area under the curve

The discrimination of the predictive model was evaluated using ROC analysis (Figure 2A), with performance parameters and the optimal cutoff determined by the Youden index, as shown in Table 4. The model demonstrated excellent discriminatory ability, with an AUC of 0.913 (95% CI: 0.876-0.95, p<0.001). At the optimal cutoff of 0.25, the sensitivity was 82.7% (95% CI: 74.1-91.2), and the specificity was 83.3% (95% CI: 77.4-89.3).

**Table 4:** Performance Parameters of Predictive Model for Early-Onset Sepsis CI: confidence intervals; ACC: accuracy; Se: sensitivity; Sp: specificity; PPV: positive predictive value; NPV: negative predictive value; PLR: positive likelihood ratio; NLR: negative likelihood ratio

Parameters	Values
Optimal cut-off	0.25
ACC %, (95% CI)	83.1 (83.0-83.2)
Se %, (95% CI)	82.7 (74.1-91.2)
Sp %, (95% CI)	83.3 (77.4-89.3)
PPV %, (95% CI)	71.3 (61.8-80.8)
NPV %, (95% CI)	90.6 (85.7-95.5)
PLR (95% CI)	4.96 (3.42-7.2)
NLR (95% CI)	0.21 (0.13-0.34)

The calibration of the model was assessed using the Hosmer-Lemeshow test, which indicated good calibration with χ²(df)=5.496 (5), p=0.358, showing no significant differences between observed and predicted values from the prognostic model. This was further confirmed by the calibration curve through a thousand bootstrap resamples, illustrated in Figure 2B. The apparent line represents the performance of the nomogram, showing good consistency between predicted and actual diagnosis probabilities of neonatal sepsis. The calibration curve revealed an overlap between the probabilities of predicted and actual diagnoses, with the bias-corrected line quite closely following the ideal line. Although some underprediction was noted at lower predicted probabilities, where the bias-corrected line is above the ideal line for predicted probabilities less than 0.3, the mean absolute error, which measures the average absolute difference between predicted and actual probabilities, was 0.041. This is lower than the 0.9 quantile of absolute error, which was 0.079. This suggests that the model is reasonably well-calibrated, with minimal deviation between predicted and observed outcomes.

DCA

**Figure 3:**
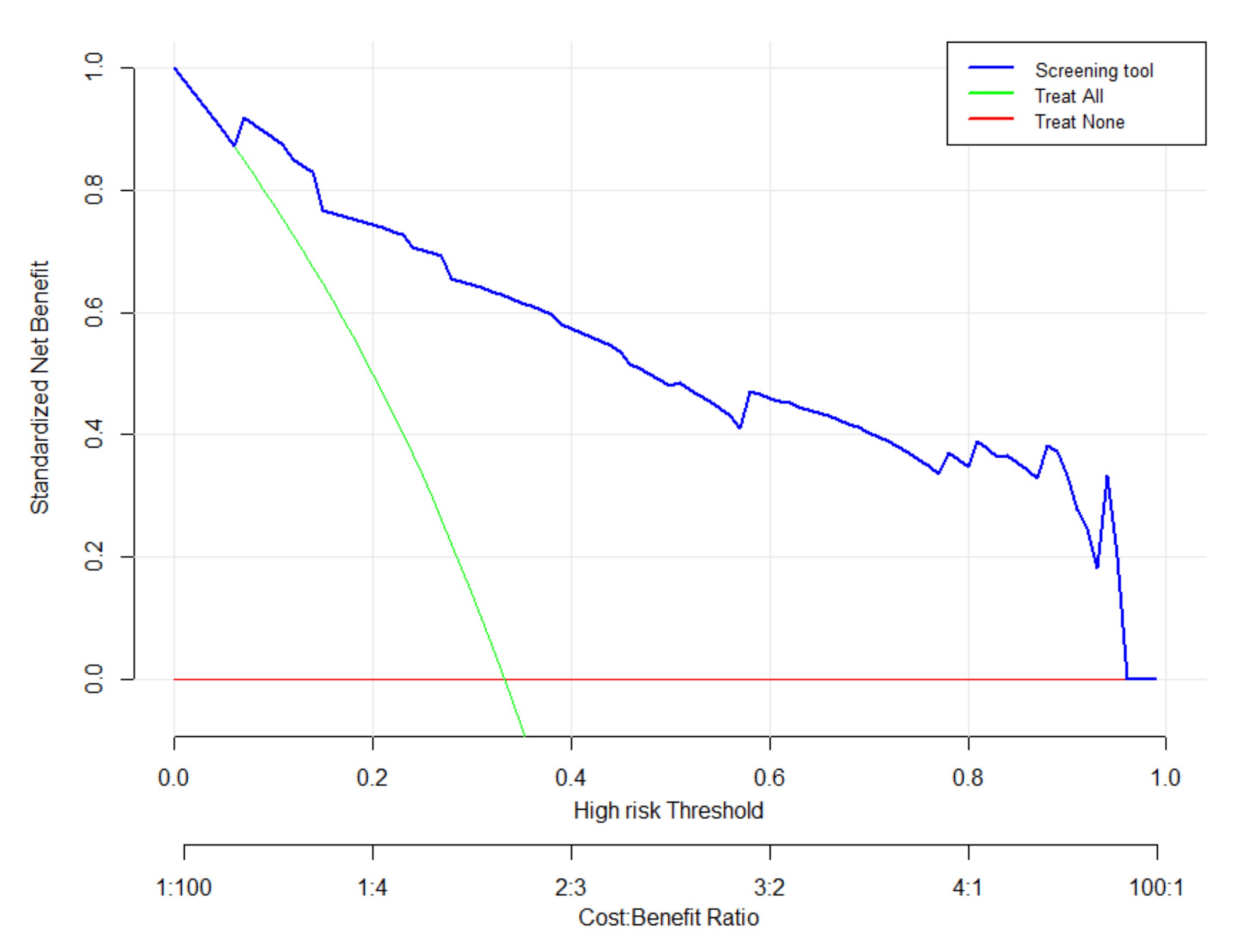
Decision Curve Analysis for Prediction of Neonatal Early-Onset Sepsis The standardized net benefit is plotted against the threshold probability. The blue line represents the decision curve of the predictive model labeled as a "Screening tool." The green line, labeled "Treat All," shows the net benefit of treating all patients, while the red line, labeled "Treat None," represents the net benefit of treating no patients, with a net profit of zero. The net gain rate is illustrated by the slope of the inverse sloping line. "Treat" signifies implementing extensive further interventions in suspected groups, including careful examination, management, and follow-up strategies.

The DCA of the model was plotted using high-risk threshold probability on the X-axis and standardized net benefit rate on the Y-axis (Figure 3). This analysis indicates that using this nomogram model to evaluate the high risk of clinical EOS in newborns yields a net clinical benefit.

The curve demonstrates that the model (blue line) provides a higher net benefit than the treat-none strategy across most threshold probability ranges (0.0-0.96) and a higher net benefit than the treat-all strategy, particularly within clinically relevant threshold probability ranges (0.07-1.0). When the threshold probability of high risk is between the blue line and the green line (0.07-1.0), the model effectively predicts the occurrence of neonatal EOS, resulting in a high net gain rate for children. This highlights the model’s ability to identify neonates at high risk for EOS, thereby facilitating timely interventions and improving clinical outcomes.

## Discussion

EOS is recognized as a leading cause of neonatal mortality, contributing to long-term neurological sequelae and imposing significant treatment costs. The diagnosis of EOS based on risk factors is increasingly being adopted, particularly in LMICs, due to its accessibility and cost-effectiveness. Implementing risk factor-based diagnostic approaches in these settings can enhance early detection and timely intervention, thereby reducing mortality rates and improving neonatal health outcomes despite limited resources [15,16]. In our study, we identified eight independent factors that can predict EOS and developed a predictive model that can serve as a screening tool for EOS. This model has been evaluated through discrimination, calibration, and DCA.

Predictors of EOS

Neonates born to mothers with genital infections in the third trimester had a 4.88-fold increased risk of developing EOS. A cohort study conducted on 2,468 neonates with mothers who had genital infections found that these infections increased the risk of neonatal sepsis by 1.6 times (adjusted relative risk (aRR)=1.60, 95% CI: 1.13-2.27) among full-term infants [17]. In our study, UTIs during pregnancy were associated with a significantly increased risk of EOS by 6.69-fold (95% CI: 2.76-16.24, p<0.001). A meta-analysis involving 1,987 neonates diagnosed with EOS and 4,814 controls reported that maternal UTIs and reproductive tract infections increased the odds of EOS by 3.61-fold (OR=3.61, 95% CI: 2.14-6.11) [18].

Our study also identified hypertensive disorders during pregnancy as an independent risk factor, which increased the risk of EOS by approximately fivefold (95% CI: 1.30-19.3, p=0.019). A Brazilian multicenter surveillance study similarly identified EOS as a common complication of hypertensive disorders during pregnancy [19]. Furthermore, chorioamnionitis is a frequent factor in predicting EOS. In this current study, chorioamnionitis was associated with a 25.61-fold increase in the odds of EOS in univariable analysis (OR=25.61, 95% CI: 3.24-202.54, p=0.002). Another study on 9,391 mother-infant pairs reported that chorioamnionitis was diagnosed in 10.3% of mothers, and clinical chorioamnionitis was associated with a 4.93-fold increase in the odds of EOS (OR=4.93, 95% CI: 1.65-14.74) [13]. The principal mechanism of chorioamnionitis is ascending bacterial infection after premature ROM, but it may also occur with intact membranes [20].

The current study has shown that meconium-stained amniotic fluid, ROM lasting more than 18 hours, and mode of delivery are significant maternal intrapartum factors associated with EOS. Meconium-stained amniotic fluid was found to significantly increase the risk of EOS by 13.89-fold (95% CI: 3.42-56.52; p<0.001). A cross-sectional study indicated that newborns from mothers without meconium-stained amniotic fluid had a lower risk of neonatal sepsis compared to those from mothers with meconium-stained amniotic fluid (adjusted odds ratio (aOR)=0.384, 95% CI: 0.152-0.968) [21]. Additionally, a systematic review and meta-analysis concluded that meconium-stained amniotic fluid was significantly associated with EOS (OR=2.9, 95% CI: 1.8-4.5) [22]. In this study, data showed that mothers with a prolonged ROM (more than 18 hours) had an approximately 12-fold increased risk of EOS (aOR=11.69, 95% CI: 1.97-69.19). A meta-analysis identified a similar association, with prolonged ROM being linked to EOS (OR=2.8, 95% CI: 1.9-4.1) [22].

Early neonatal sepsis risk factors in developing countries include inadequate antenatal care [23]. In our study, 12.4% of mothers did not gain 10 kg during pregnancy. Compared to mothers who gained more than 10 kg, those who gained less than 10 kg had an approximately six-fold higher likelihood of having a child with EOS (aOR=5.72; 95% CI: 1.62-20.22). LBW is often a result of insufficient maternal weight gain during pregnancy. Several studies have shown that important neonatal risk factors for EOS include preterm birth and LBW [22]. The current study also shows the association between insufficient maternal weight gain during pregnancy with LBW (OR=4.26; 95% CI: 1.88-9.64; p=0.001) and preterm birth less than 34 weeks (OR=4.19; 95% CI: 1.67-10.5; p=0.002). Moreover, neonates born preterm before 34 weeks of gestation had significantly higher odds of developing EOS (aOR=33.43; 95% CI: 6.78-164.87; p<0.001). Preterm infants with LBW experience infections at a rate of 3 to 10 times higher than their full-term, normal birth weight counterparts. This increased susceptibility is largely due to immune system dysfunction and the lack of maternally acquired IgG antibodies, which are typically transferred across the placenta during the later stages of pregnancy [9]. Additionally, regarding the APGAR score at one minute, there was an increased incidence of sepsis among babies with scores less than seven. Our study identified that newborns with first-minute APGAR scores of less than seven had a 20-fold higher risk of developing EOS (aOR=19.74; 95% CI: 3.56-109.55; p=0.001). A systematic review and meta-analysis also demonstrated that low APGAR scores were significantly associated with EOS (OR=2.4, 95% CI: 1.6-3.5) [22].

Predictive tool construction and validation

In the current study, the predictive model was developed using multivariable logistic regression, and the nomogram was constructed as a valuable tool in medical practice due to its ability to simplify complex predictive models into an easy-to-use graphical representation. This predictive model was evaluated based on its discrimination and calibration abilities. The model demonstrated outstanding discrimination between neonates with EOS and those without, with an AUC of 0.913 (95% CI: 0.876-0.95). A retrospective study conducted by Wu et al. also developed a risk prediction model for neonatal sepsis in group B Streptococcus(GBS)-colonized mothers, achieving an AUC of 0.713 (95% CI: 0.635-0.773) [24]. The differences in AUC values may be attributed to variations in participant characteristics, as well as the predictors and outcomes used in the models. These findings underscore the need for further research in more specific populations to enhance the generalizability and clinical applicability of predictive models for neonatal sepsis.

Good calibration was demonstrated by the Hosmer-Lemeshow test with χ^2^(df)=5.496(5), p=0.358, indicating consistent agreement between actual and predicted values from the model. Additionally, the mean absolute error suggests that the model's predictions are fairly close to the actual outcomes, with an average error of approximately 4.1%. This is lower than the 90th percentile of absolute error (7.9%), suggesting that the model consistently performs well across most of the data. These results affirm the reliability and robustness of the predictive model in estimating the risk of EOS in neonates.

The DCA evaluates the clinical usefulness and net benefit of the predictive model for neonatal EOS, comparing the net benefits of the nomogram with other strategies across a range of threshold probabilities. The DCA demonstrates the trade-off between true-positive and false-positive predictions for each strategy [25]. Additionally, the DCA curve illustrates the model's good clinical utility. The optimal metric threshold for the classification model is determined through calculation, with a maximum index of 0.25 serving as the optimal indicator threshold. When the risk of neonatal sepsis exceeds 25%, as calculated by the risk calculator, additional monitoring and attention are necessary. At a threshold probability of 0.25, the net benefit is 0.7, indicating that for every 100 neonates in the target population, 70 true positives would be identified without causing harm. The risk of 25% corresponds to odds of 1:3, meaning that using a threshold probability of 25% implies that “missing a high-risk EOS case is 3 times worse than performing an unnecessary screening” [26]. The cost-benefit ratio at this specific 0.25 threshold probability is 1:3, meaning that three false positives are acceptable for one true positive. This is justified because the predictive tool is based on available risk factors and does not cause harm or incur additional costs for newborns when used by medical staff.

Given the unique and rapid development of neonatal physiology, along with the cost-effectiveness and convenient accessibility of the predictors-based model for medical staff, we recommend using a risk threshold model of 25%, which aligns with clinical diagnostic and treatment features. This consensus underscores the importance of balancing sensitivity and specificity to optimize clinical outcomes while minimizing unnecessary interventions.

Strengths and limitations

Although this is an important initial study aimed at developing a prediction tool for screening EOS in Vietnam with an accessible nomogram and demonstrated performance, our study has several limitations. Firstly, the predictive model based on risk factors did not include infant manifestations that require clinical examination by professional pediatricians, and the diagnosis of EOS was not confirmed by positive microbial cultures. This omission may lead to some overestimation in classification, as our goal was to identify at-risk newborns immediately after delivery to enhance recognition of vulnerable groups for further care, an approach that could be acceptable in resource-limited settings. Future studies should incorporate infants' clinical signs and symptoms, as well as culture-confirmed sepsis, to provide a more comprehensive evaluation of at-risk neonates. Secondly, our model is derived from a single-center study utilizing internal validation, raising concerns about generalizability. Rigorous external validation and future multicenter studies are essential to thoroughly evaluate the prediction tool's validity. Additionally, we did not stratify based on mothers who had been treated for infections before delivery or maternal GBS status due to insufficient previous medical information, a crucial issue in Vietnam and other LMICs with unsynchronized healthcare systems. To address these limitations, future research should involve multiple centers with synchronized sharing of medical records to more accurately determine maternal risk factors and provide a comprehensive evaluation. Despite these limitations, our study provides a foundational step toward improving EOS screening in Vietnam.

## Conclusions

This study underscores the critical importance of early detection and intervention for neonatal EOS in Vietnam, where routine microbial cultures are impractical, by developing a predictive model based on key maternal and neonatal risk factors. The nomogram, demonstrating excellent discrimination and good calibration, facilitates the prompt screening of suspected neonates. While the model requires external validation and the inclusion of more comprehensive clinical data, it represents a significant advancement in managing EOS and has the potential to reduce sepsis-related morbidity and mortality in resource-limited settings. Future research should focus on multi-center studies and broader data integration to enhance the model’s robustness and applicability.
